# Microsurgical training curriculum in a gynecological breast cancer center: a benefit for patients and surgeons?

**DOI:** 10.1007/s00404-023-07198-z

**Published:** 2023-08-29

**Authors:** Georg Schmidt, Theresa Mayo, Stefan Paepke, Marion Kiechle, Daniel Müller

**Affiliations:** Department of Gynecology and Obstetrics, Klinikum Rechts Der Isar and Comprehensive Cancer Center (CCCTUM), Technical University Munich, Munich, Germany

**Keywords:** Oncoplastic breast surgery, Breast reconstruction, Microsurgery, Deep inferior epigastric perforator flap, Surgical education

## Abstract

**Purpose:**

Autologous breast reconstruction improves patient satisfaction and quality of life after mastectomy. In Germany, free flap surgery and implant-based reconstruction is usually separate between reconstructive surgery and gynecology. Cooperation between the specialist disciplines and implementation of microsurgery into breast surgeon training could enhance surgical treatment for breast cancer patients. This evaluation is intended to demonstrate the learning progress within a microsurgical training program and the complication rate in relation to microsurgical experience.

**Methods:**

At the breast cancer center at Klinikum rechts der Isar, TU Munich, a three-stage training program for autologous breast reconstruction and microsurgery for gynecological breast surgeons was developed. Between 2019 and 2022, 74 women received autologous free flap breast reconstruction by a consistent team consisting of a gynecological surgeon in training and an expert microsurgeon. Peri- and postoperative data were collected to analyze the feasibility and safety of a microsurgical training in gynecology.

**Results:**

Within the training, operative steps of free autologous breast reconstruction were increasingly taken over by the gynecological surgeon in training. The analysis showed a decrease in operating times with consistently low complication rates during the training.

**Conclusion:**

This study demonstrated that a training in free autologous breast reconstruction for gynecological surgeons is safely feasible through close cooperation between gynecological and reconstructive surgery.

## What does this study add to the clinical work

**Table:** 

Through a structured training program, microsurgical techniques can be implemented within a gynecological breast cancer center. Expanding the surgical portfolio can lead to improved patient care without an increase in complications.	

## Introduction

With approximately 69.400 new cases in Germany in 2018, breast cancer is the most common cancer in women [1]. When first diagnosed, 93–94% of patients present with an UICC stage < IV. Breast surgery is a major part of therapy for these patients. Depending on tumor size, multicentricity, breast size, genetic factors and the patient’s wishes, breast-conserving therapy can be performed in about 75% of cases. For about a quarter of patients, a mastectomy of the affected breast is indicated. Based on the number of new cases in 2018 and a mastectomy rate of 25%, this would affect more than 16.000 patients in Germany. Preoperatively, these patients need to be counseled concerning reconstructive options at a certified breast center. Depending on the surgical procedure, reconstruction can be implant-based or use autologous tissue. (Post-neo-) adjuvant chemotherapy and adjuvant irradiation must be considered in the consultation and surgical planning. In Germany, implant-based reconstruction is usually performed by a gynecological surgeon, while autologous reconstruction is usually performed by a plastic and reconstructive surgeon.

Women are usually advised by gynecologists at a certified breast center. This could explain the rate of implant-based reconstructions being as high as 70–80% [2]. The high number of implant-based reconstructions is surprising, as autologous breast reconstruction is associated with significantly higher satisfaction and sexual and emotional well-being [3–5]. In the UK health service, there has been a decrease in autologous breast reconstruction and an increase in implant-based reconstruction in recent years. The higher costs and poorer widespread availability of autologous reconstruction are cited as possible reasons [4].

During the last years, the Department of Gynecology and Obstetrics at Klinikum rechts der Isar, Technical University of Munich, has aimed to widen their range of breast reconstruction methods offered in order to be able to grant their patients every form of reconstruction available. An experienced plastic surgeon has been permanently integrated into the team of the certified breast center. Patients are informed about the surgical options at an early stage after diagnosis and can choose their preferred way of breast reconstruction. In addition, a training concept was designed to train gynecological breast surgeons in breast reconstruction with free flap surgery. The training concept is presented below. The main objectives of this study were to evaluate how the learning curve in microsurgical training progresses and whether complications increase with the involvement of gynecologic surgeons in microsurgical procedures. The target parameters were defined as the number of procedures per training level, the duration of the operations and the number of complications associated with the microsurgical procedure. This study could be a model for other breast center to strengthen the collaboration with plastic and reconstructive surgery and form interdisciplinary teams for autologous breast reconstruction. The aim is to improve the surgical offers for patients suffering from breast cancer and to optimize the interdisciplinary work between gynecology and reconstructive surgery.

## Material and methods

Patients who underwent autologous breast reconstruction using free flaps at the interdisciplinary breast center of the Department of Gynecology and Obstetrics, Klinikum rechts der Isar, between January 2019 and January 2022 were included in the analysis. A retrospective data analysis was performed. The project was reviewed by the ethics committee of Technical University of Munich. The number of the approval is 2023-113-S-SR. Data on surgery and breast cancer therapy were taken from the SAP^®^ hospital information system. The evaluation was anonymized. IBM^®^ SPSS^®^ Statistics version 26 was used for the descriptive statistical analysis.

## Results

### Structure of the training program

At the beginning of the project, a curriculum was developed for the microsurgical training of a qualified senior breast surgeon according to OnkoZert (Deutsche Krebsgesellschaft). The concept was developed in cooperation with an experienced senior physician in plastic and reconstructive surgery. The requirement for the training was the qualification as a certified breast surgeon with surgical expertise in breast-conserving surgery, oncoplastic surgery and implant-based breast reconstruction. The board-certified plastic surgeon has experience in over 2000 microsurgical procedures and in more than 500 free flap breast reconstructions. In addition to breast reconstructions, hand surgery and free flap surgery to cover defects on the entire body are part of the trainer’s portfolio. As an instructor in such a training program, we recommend working with a reconstructive surgeon, who routinely and independently performs free flaps and is familiar with the management of complications due to a high number of microsurgical interventions. The trainee’s requirements should include experience of at least 150 breast-conserving operations and at least 50 mastectomies with implant-based reconstruction.

Once the gynecological surgeon has learned the skills from one level of training, the next level of training follows. No minimum number of interventions per level has been defined. The learning progress is assessed by the senior plastic and reconstructive surgeon.

Level 1 of the program should initially focus on continuous assistance during free flap surgery. In the case of primary reconstruction, the mastectomy is performed by the trainee breast surgeon. Preparatory steps, such as the preparation of the internal mammary artery (IMA), are carried out by the gynecologist. Working with magnification is also practiced. Microanastomosis and harvesting of the flap are performed by the plastic surgeon with the assistance of the breast surgeon.

The abdominoplasty for abdominal wall closure and the closure on the thigh after harvesting the Transverse Myocutaneous Gracilis Flap (TMG) are gradually taken over by the trainee. Postoperative management and assessment of flap perfusion is part of the training. The trainee attends microsurgical courses to practice microsurgical suturing techniques on training models and on live rat models. The microsurgical training on 3-D and rat models focuses on microscopic field manipulation, experience with the tissue of the vessels and knot tying.

In level 2, the trainee begins to take over microanastomosis and preparation of the perforator vessels. The veins are anastomosed with a coupler system and the arteries are sutured. These steps take place under the supervision of the senior surgeon, who can intervene at any time. Difficult surgical steps, e.g., after irradiation, continue to be performed by the senior surgeon.

**Fig. 1:**
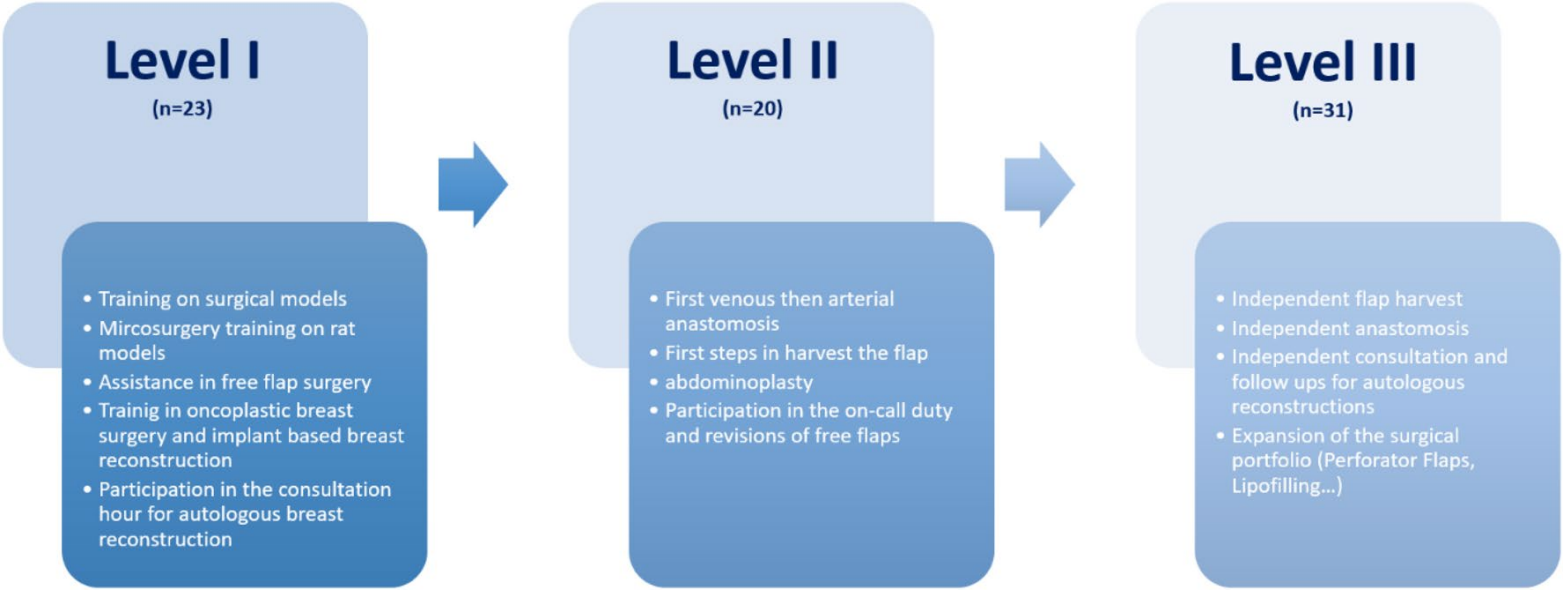
Structure of the training curriculum

As soon as the microsurgical techniques are confidently mastered, in level 3 both flap elevation and microanastomosis are performed by the breast surgeon in training, further assisted by the senior surgeon. Harvesting the flap and the preparation of the IMA can now be done in parallel to accelerate the operation. In addition to free flap surgery, other techniques for breast surgery are trained, such as pedicled perforator flap surgery to cover volume defects (AICAP, LICAP, TAP flap, etc.). The patients are advised about these reconstructive techniques in the consultation hours prior to surgery (Fig. 1).

### Data analysis

In the observation period from January 2019 to January 2022, 74 patients underwent breast reconstruction with autologous tissue by the same team of plastic surgeon and breast surgeon. The average age of the patients was 51 years. The youngest patient was 26 years, the oldest patient 80 years old. In 62.2% (*n* = 46), the breast reconstruction was immediate, and in 37.8% (*n* = 28), the breast reconstruction was delayed. Depending on patient weight, previous operations, vascular status, anatomical conditions and the patient’s wishes, the type of reconstruction was either a DIEP flap (Deep Inferior Epigastric Perforator Flap), free muscle-sparing TRAM flap (Transverse Rectus Abdominis Myocutaneous Flap) or TMG flap (Transverse Myocutaneous Gracilis Flap). Fifty-one patients were reconstructed using DIEP flap, 12 patients using muscle-sparing TRAM flap and 11 women using TMG flap. Among the 51 DIEP flaps, two women received a bilateral DIEP flap.

After 23 operations (31.1%) in training level 1, 20 operations (27%) were performed in level 2. In the remaining 31 (41.9%) cases, the breast surgeon performed flap harvest and microanastomosis himself.

The median operation time between incision and end of suture was 5:42 h (342 min). The longest operation lasted 10:10 h (610 min), the shortest 3:57 h (237 min).

During training level 1, the median operation time was 6:10 h (370 min), in level 2 5:24 h (324 min) and in level 3 5:33 h (333 min) (Table 1). There was a bilateral DIEP operation each in level 1 and level 2. However, there is no overall increase in operating time due to the breast surgeon’s training in increasingly complex surgical steps.

**Table 1:** Median operative time

Operative time			
Level	Mean operative time (hours)	*N*	Standard deviation (hours)
I	6:10	23	1:16
II	5:24	20	0:48
III	5:33	31	0:56
Overall	5:42	74	1:03

All complications were documented, regardless of whether a surgical revision was necessary.

Major complications include complete flap loss and revisions for thrombosis of the flap pedicle.

There was one loss of the flap during hematoma evacuation and tear of the pedicel (1.4%). Two patients underwent revision surgery due to venous thrombosis within the first 24 h after surgery (2.7%). In each case, the thrombosis was removed without further complications (Table 2)*.*

**Table 2:** Major complications

Major complications	Level I *n* = 1	Level II *n* = 0	Level III *n* = 2	Overall *n* = 3/74 (4.1%)
Total flap loss	*n* = 0	*n* = 0	*n* = 1	*n* = 1 (1.4%)
Revision of the pedicle/anastomosis	*n* = 1	*n* = 0	*n* = 1	*n* = 2 (2.7%)

Among the 74 patients, postoperative minor complications at the chest wall occurred in eight cases (10.8%) within the first six weeks after surgery.

Four women developed impaired wound healing and were treated conservatively. Four women with hematoma or seroma were all treated conservatively (Table 3).

**Table 3:** Minor complications with conservative treatment

Minor complications conservative treatment	Level I *n* = 2	Level II *n* = 4	Level III *n* = 2	Overall *n* = 8/74 (10.8%)
Wound healing disorder	*n* = 1	*n* = 2	*n* = 1	*n* = 4 (5.4%)
Hematoma/seroma	*n* = 1	*n* = 2	*n* = 1	*n* = 4 (5.4%)

Revision of the abdomen was necessary in two cases, one for hernia repair and another due to wound infection (one in Level 2, one in Level 3). Three women (one in each group) developed partial necrosis at Level IV of the flap, resulting in necrosectomy. The necrosectomy took place within the resection of the skin paddle for flap monitoring and final fitting of the flap after 5–7 days after primary surgery.

## Discussion

Autologous reconstruction of the breast is not part of residency training in gynecology and obstetrics in Germany. The catalog of requirements for certification as a senior breast surgeon by the German Cancer Society does not require expertise in autologous breast reconstruction [6]. Another way of certifying advanced expertise in reconstructive gynecological surgery is to be certified as a Breast Surgeon by the Arbeitsgemeinschaft für ästhetische, plastische und wiederherstellende Operationsverfahren in der Gynäkologie e.V. (AWOgyn). The certification has high requirements. Seventy-five breast reconstructions with autologous tissue must have been performed in the past five years [7]. However, free flap surgery with vascular anastomosis is not explicitly required. It can be assumed that the majority of the required reconstructions are covered by latissimus dorsi flaps or pedicled TRAM flaps. So far, there is no training program in Germany for autologous breast reconstruction with free flaps in gynecology.

The German Society of Plastic, Reconstructive and Aesthetic Surgeons (DGPRÄC) offers certification as a breast surgeon and as a center specializing in microsurgical breast reconstruction [8]. The requirements are 50 shape-changing breast operations including 20 microsurgical operations per year. Only residents in plastic surgery have access; gynecologists cannot obtain this qualification.

Our data analysis shows that microsurgical training can also be safe and effective in gynecology. The microsurgical procedures required by the DGPRÄC were exceeded in our breast center every year. Through numerous implant-based breast reconstructions, the requirement for shape-changing operations is also easily achieved.

However, the lack of microsurgical training within the gynecological residency in contrast to plastic surgery must be emphasized. The missing experience with free flap surgery in gynecology can be a disadvantage in the therapeutic management of breast cancer patients. Whether a patient is presented to a plastic surgeon preoperatively depends most certainly on the depth of cooperation between a certified breast center and a reconstructive unit.

Microsurgical training in gynecology could improve the surgical therapy. In microsurgery, there is a long learning curve due to its complex nature and the training demands a lot of patience from the surgeons in training. In plastic and reconstructive surgery, an increasing number of young doctors are migrating to more profitable aesthetic fields. A survey of 708 plastic surgeons in the USA showed that about one-third of the respondents had signs of burn-out [9]. Primarily reconstructive work and on-call duties were found to be significant risk factors for developing a burn-out. Another survey among US microsurgeons confirms the poorer professional satisfaction in the group of reconstructive surgeons [10]. Reconstructive oral and maxillofacial surgeons also show a higher workload and dissatisfaction with their working conditions [11].

With such disillusioning data, does it make sense at all to integrate microsurgery into gynecology? By sharing the surgical steps during free flap surgery, the workload for the plastic surgeon can be reduced. An analysis of more than 8000 autologous breast reconstructions showed that microsurgical reconstruction performed by two surgeons simultaneously does not increase complications [12]. Another analysis shows that operating times and inpatient stays are lower for autologous breast reconstruction performed by two surgeons simultaneously than for operations by a single microsurgeon [13]. While this study compared surgeries by experienced microsurgeons, our training program also shows that surgeries performed by an experienced microsurgeon and a microsurgeon-in-training can lead to a shortened surgery time without increasing complication rates.

Besides shorter operation times, an expansion of the microsurgical team could lead to higher job satisfaction. In the case of long operations, work steps can be shared between the two surgeons and on-call duties and complication management do not have to be borne by a single surgeon. This could prevent work-related overload as described above.

In gynecology, there are no established and structured training programs for microsurgery so far. However, the form and scope of microsurgical training also varies significantly in plastic and reconstructive surgery. A survey of plastic surgeons in 60 American centers showed the heterogeneity in microsurgical training. Training includes exercises on in vitro and in vivo models, some virtual practice opportunities and assisting in the operating room. A large proportion of respondents expressed a desire for a structured curriculum at their training center [14].

A meta-analysis of different training programs recommends training on microsurgical models before training on patients [15]. In our curriculum, the trainee gains experience in the operation room in addition to training on models in an early stage of microsurgical training.

## Summary

A three-stage microsurgical training program for gynecologic breast surgeons was developed at the certified breast cancer center of the Klinikum rechts der Isar of the Technical University of Munich to learn all steps of autologous breast reconstruction. Between 2019 and 2022, 74 patients underwent surgery performed by an experienced reconstructive surgeon and a gynecologic breast surgeon. Gradually, microsurgical and other surgical steps were adopted from the gynecologic side. Data analysis showed a decrease in operative time and a consistently low complication rate over the course of the curriculum. These data support expansion of microsurgical training in gynecology.

The goal of increasing expertise in autologous breast reconstruction among gynecologists is to improve surgical care for patients with breast cancer or with mutations in a high-risk gene. The goal should not be to compete with reconstructive surgeons for autologous breast reconstruction. Rather, interdisciplinary collaboration should be strengthened and patients should be cared for in certified breast centers across disciplines with the best possible expertise. If gynecologists come into contact with autologous breast reconstruction early in their surgical training, they will be able to advise patients according to their needs and recommend autologous tissue reconstruction more frequently, which will also benefit plastic and reconstructive surgery.

## Limitation

The evaluation of the training program was retrospective and monocentric. Further prospective and multicentric projects are desirable to further evaluate microsurgical training models.

## Data Availability

The data that support the findings of this study are available from the corresponding author, G.S., upon reasonable request. No data that violates the personal rights of patients will be shared.
